# Calcium and adenosine triphosphate control of cellular pathology: asparaginase-induced pancreatitis elicited via protease-activated receptor 2

**DOI:** 10.1098/rstb.2015.0423

**Published:** 2016-08-05

**Authors:** Shuang Peng, Julia V. Gerasimenko, Tatiana Tsugorka, Oleksiy Gryshchenko, Sujith Samarasinghe, Ole H. Petersen, Oleg V. Gerasimenko

**Affiliations:** 1 Cardiff School of Biosciences, Cardiff University, Cardiff CF10 3AX, Wales, UK; 2 Department of Pathophysiology, Medical College, Jinan University, Guangzhou 510632, People's Republic of China; 3 Bogomoletz Institute of Physiology, Kiev 01024, Ukraine; 4 Great Ormond Street Hospital for Children NHS Foundation Trust, Great Ormond Street, London WC1N 3JH, UK; 5 Systems Immunity Research Institute, Cardiff University, Cardiff CF14 4XN, Wales, UK

**Keywords:** calcium, adenosine triphosphate, asparaginase, pancreatitis, leukaemia, calcium channels

## Abstract

Exocytotic secretion of digestive enzymes from pancreatic acinar cells is elicited by physiological cytosolic Ca^2+^ signals, occurring as repetitive short-lasting spikes largely confined to the secretory granule region, that stimulate mitochondrial adenosine triphosphate (ATP) production. By contrast, sustained global cytosolic Ca^2+^ elevations decrease ATP levels and cause necrosis, leading to the disease acute pancreatitis (AP). Toxic Ca^2+^ signals can be evoked by products of alcohol and fatty acids as well as bile acids. Here, we have investigated the mechanism by which l-asparaginase evokes AP. Asparaginase is an essential element in the successful treatment of acute lymphoblastic leukaemia, the most common type of cancer affecting children, but AP is a side-effect occurring in about 5–10% of cases. Like other pancreatitis-inducing agents, asparaginase evoked intracellular Ca^2+^ release followed by Ca^2+^ entry and also substantially reduced Ca^2+^ extrusion because of decreased intracellular ATP levels. The toxic Ca^2+^ signals caused extensive necrosis. The asparaginase-induced pathology depended on protease-activated receptor 2 and its inhibition prevented the toxic Ca^2+^ signals and necrosis. We tested the effects of inhibiting the Ca^2+^ release-activated Ca^2+^ entry by the Ca^2+^ channel inhibitor GSK-7975A. This markedly reduced asparaginase-induced Ca^2+^ entry and also protected effectively against the development of necrosis.

This article is part of the themed issue ‘Evolution brings Ca^2+^ and ATP together to control life and death’.

## 1. Background

The importance of Ca^2+^ for the control of secretion has been known for a long time [1]. However, it was only through a detailed analysis—using permeabilized adrenal chromaffin cells—that the intracellular requirements for exocytotic secretion were clarified [2]. This work established that secretion occurs when the intracellular free Ca^2+^ concentration ([Ca^2+^]_i_) increases from the basic level (around 100 nM) to the low μM range if, and only if, Mg-adenosine triphosphate (ATP; 0.5–5 mM) is present [2]. Under physiological conditions, the rise in [Ca^2+^]_i_ that initiates secretion from intact chromaffin cells, as well as nerve endings, is owing to Ca^2+^ entering the cells through voltage-gated Ca^2+^ channels [2–4]. This is, however, fundamentally different from what happens in the electrically non-excitable exocrine gland cells [5,6], where neurotransmitter- or hormone-evoked enzyme and fluid secretion are initiated by release of Ca^2+^ from the endoplasmic reticulum (ER) [7], mediated by the intracellular messenger inositol 1,4,5-trisphosphate (IP_3_) [8]. The primary intracellular Ca^2+^ release is followed by secondary Ca^2+^ entry from the extracellular solution, and this Ca^2+^ influx does not occur through voltage-gated Ca^2+^ channels, but via Ca^2+^ release-activated Ca^2+^ (CRAC) channels [9].

The physiologically relevant Ca^2+^ signals that control secretion in the pancreatic acinar cells occur as repetitive local [Ca^2+^]_i_ spikes in the apical secretory granule region [10,11], owing to primary Ca^2+^ release from thin strands of ER penetrating into this region [12,13], which is dominated by zymogen granules (ZG). These ER strands are fully functionally connected (ER tunnels) to the principal ER store dominating the basal region [12,13]. The IP_3_ receptors (IP_3_Rs) are concentrated in the apical area [11,14,15] and the functional ER tunnels effectively allow Ca^2+^ to be mobilized from the main basal store into the apical secretory granule area [12]. Plasma membrane Ca^2+^ pumps, concentrated in the apical (secretory) membrane [16], extrude Ca^2+^ from the cell during each Ca^2+^ spike, and compensatory Ca^2+^ entry and uptake into the ER are therefore necessary. This occurs through conventional [17] CRAC channels [9] localized in the baso-lateral membrane [12,18], and Ca^2+^ entering through these channels is immediately taken up into the ER by powerful Ca^2+^ pumps in the ER membrane [12]. The physiological apical Ca^2+^ release via IP_3_Rs in that region results in apically confined [Ca^2+^]_i_ spikes owing to the mitochondrial firewall separating the ZG-containing part of the cell from the ER-dominated baso-lateral part [19]. During each apical Ca^2+^ spike, Ca^2+^ is taken up into the peri-granular mitochondria and then slowly released [13,20]. This mitochondrial Ca^2+^ uptake, activating several Ca^2+^-sensitive dehydrogenases in the Krebs cycle [21], results in an increased cytosolic ATP level, despite the increased ATP utilization [22]. This is helpful for powering the exocytotic secretory process.

Whereas the Ca^2+^ release evoked by the neurotransmitter acetylcholine (ACh) is primarily mediated by IP_3_ acting on IP_3_Rs [23,24], the Ca^2+^ release elicited by physiological concentrations of the hormone cholecystokinin (CCK) is primarily mediated by the Ca^2+^-releasing messenger nicotinic acid adenine dinucleotide phosphate (NAADP) [25–27]. The action of NAADP depends on functional ryanodine receptors (RyRs) and two-pore channels (TPCs) and also involves acid Ca^2+^ stores [27]. Physiological Ca^2+^ spiking, irrespective of whether it is evoked by ACh or CCK, depends on both functional IP_3_Rs and RyRs [13,25].

Although the acinar cells in the pancreas are quantitatively dominant and perform the most crucial physiological role in the exocrine pancreas, by synthesizing and secreting the digestive (pro-) enzymes that are essential for the digestion of food in the intestine, there are other cells that need to be considered. The acinar cells secrete fluid together with the enzymes [5], but most of the fluid carrying the enzymes into the gut is produced by the duct cells, principally regulated by secretin-evoked cyclic AMP formation rather than by Ca^2+^ signals [28,29]. The acinar–ductal system is functionally integrated and regulation of one cell type has influence on the other [30]. The physiological role of the more recently discovered peri-acinar stellate cells has not yet been fully clarified, but they generate substantial Ca^2+^ signals in response to concentrations of bradykinin that have been measured in plasma during exercise and pancreatitis [31,32]. They do not respond to the principal controllers of acinar cell function, namely ACh or CCK [31,32].

In the normal digestion process, the inactive acinar pancreatic pro-enzymes, which are secreted by exocytosis into the acinar lumen, are carried into the gut by acinar and ductal fluid secretion and then activated in the gut [33]. In acute pancreatitis (AP), a potentially fatal human disease, the inactive pancreatic pro-enzymes become active enzymes inside the acinar cells, digesting the pancreas and its surroundings. The main causes of AP are biliary disease (gallstone complications) and alcohol abuse [33]. More than 20 years ago, it was proposed that AP is essentially a disease brought about by excessive cytosolic Ca^2+^ signals [34] and since then a substantial amount of evidence in favour of this hypothesis has accumulated [35–38]. Pathological stimuli—for example, combinations of alcohol and fatty acids or bile acids—can evoke excessive release of Ca^2+^ from internal stores followed by excessive Ca^2+^ entry through store-operated CRAC channels, resulting in sustained global elevations of [Ca^2+^]_i_ [38]. This causes intracellular protease activation [38] and excessive mitochondrial Ca^2+^ uptake, leading to opening of the mitochondrial permeability transition pore (MPTP) [20,39]. The MPTP opening causes depolarization of the inner mitochondrial membrane, resulting in failure of the normal mitochondrial ATP production [20,39]. The lack of ATP prevents the acinar cells from disposing of the excess Ca^2+^ in the cytosol and, in combination with the abnormal intracellular protease activity, this leads to necrosis. It is the acinar necrosis that generates the damaging inflammatory response [39–42]. Since the primary pathological event in AP is the excessive and sustained [Ca^2+^]_i_ elevation and as this depends on excessive Ca^2+^ entry through CRAC channels, it would be logical, as a therapy, to target these Ca^2+^ entry channels. Gerasimenko *et al.* [9,38] demonstrated, in experiments on isolated mouse acinar cell clusters, that the intracellular protease activation and necrosis evoked by fatty acid ethyl esters—non-oxidative combinations of ethanol and fatty acids, which are important mediators of alcohol-related pancreatitis [33]—could be effectively inhibited by specific pharmacological blockade of CRAC channels. These results have recently been confirmed *in vivo*, in three different mouse models of AP [43], giving hope that CRAC channel blockade may become the first rational and effective therapy against AP [44].

As both alcohol-related and bile-induced AP are owing to toxic Ca^2+^ signal generation, it seems possible that all types of AP are Ca^2+^ toxicity diseases. We were therefore interested in exploring the mechanism underlying pancreatitis caused by a side-effect of l-asparaginase, which is used for the treatment of acute lymphoblastic leukaemia (ALL). l-asparaginase is a well-known anticancer agent effective against lymphoid malignancies. Since 1971, it has been an essential element in the successful treatment of ALL, the most common type of cancer affecting children [45,46]. However, asparaginase treatment can result in AP (asparaginase-associated pancreatitis (AAP). This occurs in about 5–10% of cases and AAP is the most frequent cause of discontinuing the asparaginase treatment [47–50]. The mechanism underlying the development of AAP has so far not been explored [48].

The aim of the present study was to define the mechanism underlying AAP and then to identify potential steps for therapeutic intervention. As outlined above, the history of our path to the current understanding of AP shows that studies on isolated cells or cell clusters have been enormously helpful and our approach has therefore been to study, for the first time, the effects of asparaginase on isolated mouse acinar cells or cell clusters. As *in vitro* results concerning the effects of, for example, fatty acid ethyl esters or bile acids [9,38,43] have turned out to be excellent predictors of the outcome of studies of real AP *in vivo* [39,43,51], acute studies on isolated cells are a natural starting point for investigations of the mechanism of AAP.

Our results show that asparaginase acts on protease-activated receptor 2 (PAR2) to evoke sustained elevations of [Ca^2+^]_i_ owing to release of Ca^2+^ from internal stores, followed by Ca^2+^ entry from the extracellular solution. The sustained [Ca^2+^]_i_ elevation reduces ATP formation. These effects can be markedly reduced by specific pharmacological blockade of CRAC channels, which also markedly reduces the extent of necrosis.

## 2. Results

### (a) Asparaginase increases [Ca^2+^]_i_ in pancreatic acinar cells

Pancreatitis-inducing agents, combinations of ethanol and fatty acids or bile acids, are able to elevate [Ca^2+^]_i_ in isolated acinar cell clusters causing intracellular Ca^2+^ overload. This is owing to Ca^2+^ release from internal stores triggering excessive store-operated Ca^2+^ entry [9,38]. To identify the mechanism of action of asparaginase, we therefore started out by testing the effects of asparaginase on [Ca^2+^]_i_ over a wide concentration range. One of the challenges inherent in this approach is that the time course needed for a study on normal freshly isolated cells is quite different from that of the development of AAP in the clinical situation. AAP typically develops several days after several injections (over many weeks) of asparaginase [49], whereas studies on isolated cells in the laboratory require observations of the effects of asparaginase within hours. In the present study, we have worked with the lowest concentration of asparaginase that reliably evoked cellular changes that are similar to those previously found to be associated with AP initiated by alcohol metabolites or bile acids.

**Figure 1.**
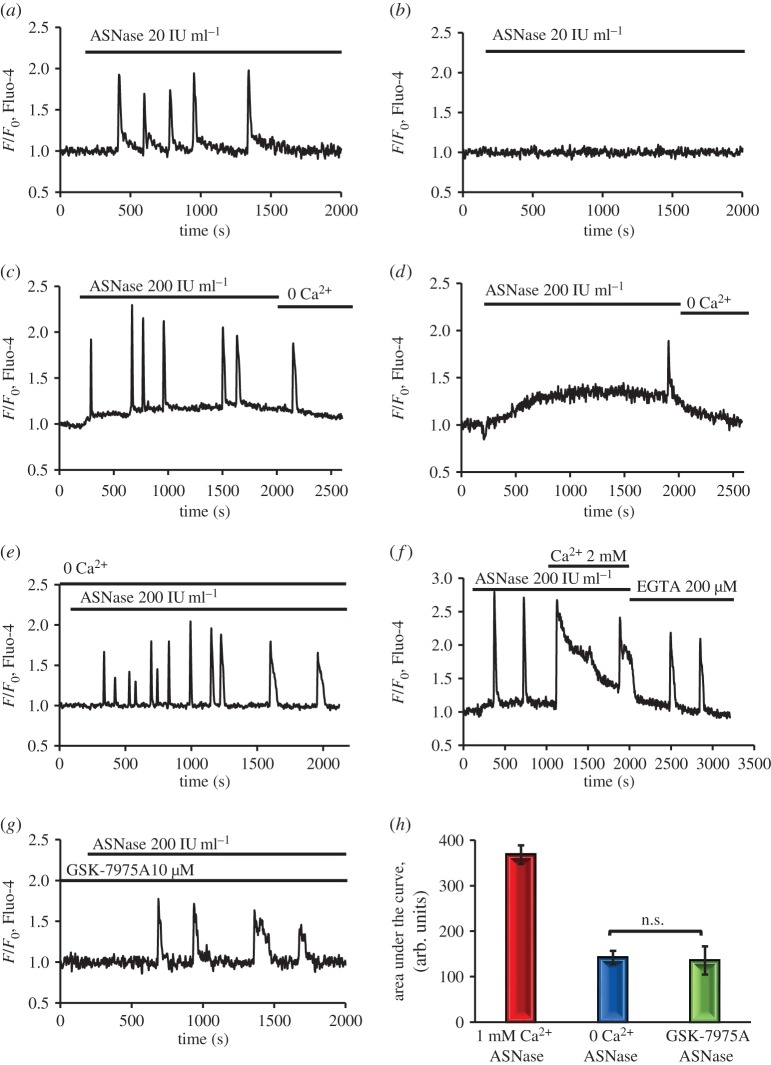
Asparaginase (ASNase) induces cytosolic Ca^2+^ signals in pancreatic acinar cells. A low concentration of asparaginase (20 IU ml^−1^) induces repetitive Ca^2+^ spikes in a minority (9 out of 42) of experiments (*a*), but elicits no response in the majority (33 out of 42) of cases (*b*). A higher concentration of asparaginase (200 IU ml^−1^) elicits an elevated [Ca^2+^]_i_ plateau in practically all cases (52 out of 55), often with repetitive Ca^2+^ transients on top of the plateau (43 out of 55) as shown in the representative trace (*c*). An elevated [Ca^2+^]_i_ plateau is the main type of response to asparaginase (200 IU ml^−1^; 52 out of 55), but in some cases (12 out of 55) with no or very few spikes (*d*). The sustained [Ca^2+^]_i_ elevation depends on the continued presence of Ca^2+^ in the external solution (*c*,*d*). In the absence of external Ca^2+^, there is no elevated [Ca^2+^]_i_ plateau (*e*, *n* = 28). When the external Ca^2+^ concentration is increased from 1 to 2 mM, there is a marked transient [Ca^2+^]_i_ rise and a continuing elevated [Ca^2+^]_i_ plateau (*f*). When external Ca^2+^ is subsequently removed and a Ca^2+^ chelator (EGTA) added, the plateau gradually disappears (*f*; *n* = 18). In the presence of the CRAC channel blocker GSK-7975A (10 µM), asparaginase (200 IU ml^−1^) evokes repetitive Ca^2+^ spikes without an elevated [Ca^2+^]_i_ plateau (*g*, *n* = 32). Comparison of the integrated Ca^2+^ signals (‘area under the curve’ from start of the Ca^2+^ signal until 1800 s later), in the presence of 1 mM external Ca^2+^, in the absence of external Ca^2+^ and in the presence of 1 mM external Ca^2+^ with the addition of 10 µM GSK-7975A, shows that the absence of external Ca^2+^ (*n* = 28) or the addition of the CRAC blocker GSK-7975A (*n* = 32) significantly (*p* < 0.0001 in both cases) reduced the responses to asparaginase (200 IU ml^−1^; *h*). Bars represent mean ± s.e.m. (Online version in colour.)

We found that only in some cells did a low concentration of asparaginase (20 IU ml^−1^) induce [Ca^2+^]_i_ oscillations (9 out of 42 cells). Figure 1*a* shows a representative positive trace with repetitive Ca^2+^ spikes induced by 20 IU ml^−1^, whereas figure 1*b* represents the more typical negative response (33 out of 42 cells), in which the same concentration of asparaginase failed to cause any change in [Ca^2+^]_i_. A higher concentration of asparaginase (200 IU ml^−1^) elicited repetitive [Ca^2+^]_i_ oscillations, mostly on top of a sustained elevated [Ca^2+^]_i_ (43 out of 55 cells; figure 1*c*). The development of a sustained [Ca^2+^]_i_ elevation has previously been shown to be a distinctive characteristic of [Ca^2+^]_i_ changes induced by pathological concentrations of alcohol metabolites or bile acids [9,38], and we found that an elevated [Ca^2+^]_i_ plateau, although often small, was seen in the vast majority of cells (52 out of 55) stimulated by asparaginase (200 IU ml^−1^). In some cases (12 out of 55), there were no, or very few, spikes superimposed on the elevated [Ca^2+^]_i_ plateau (figure 1*d*).

### (b) The asparaginase-elicited sustained increase in [Ca^2+^]_i_ depends on the presence of external Ca^2+^

Removal of external Ca^2+^ always terminated the elevated [Ca^2+^]_i_ plateau (figure 1*c*,*d*), but did not significantly (*p* > 0.27) change the amplitudes of the asparaginase-induced Ca^2+^ spikes (*n* = 28) within the time frame of our experiments (figure 1*e*). Ca^2+^ entry clearly plays an important role in the formation of the asparaginase-induced elevated [Ca^2+^]_i_ plateau and the role of Ca^2+^ entry was further demonstrated by increasing the external Ca^2+^ concentration to 2 mM during asparaginase stimulation, which caused a marked and sustained increase in [Ca^2+^]_i_, (*n* = 18; figure 1*f*). Subsequent removal of external Ca^2+^ and addition of the Ca^2+^ chelator EGTA (200 µM) abolished the elevated [Ca^2+^]_i_ plateau, whereas the Ca^2+^ oscillations continued for some time (figure 1*f*).

It has previously been shown that the excessive Ca^2+^ entry into pancreatic acinar cells induced by alcohol metabolites or bile acids, as well as their pathological effects, can be markedly inhibited by the CRAC channel blockers GSK-7975A and CM-128 [9,43,52]. We have therefore tested the effect of CRAC blockade (GSK-7975A, 10 µM) on the asparaginase-induced sustained [Ca^2+^]_i_ elevation and found that it was abolished in the presence of the inhibitor, although repetitive Ca^2+^ spiking was still observed within the time frame of our experiments (figure 1*g*, *n* = 32). Figure 1*h* summarizes the degree of inhibition, caused by removal of external Ca^2+^ or by GSK-7975A, of the integrated Ca^2+^ signal evoked by asparaginase.

### (c) Asparaginase-elicited Ca^2+^ release involves inositol 1,4,5-trisphosphate receptors and ryanodine receptors as well as nicotinic acid adenine dinucleotide phosphate signalling

**Figure 2.**
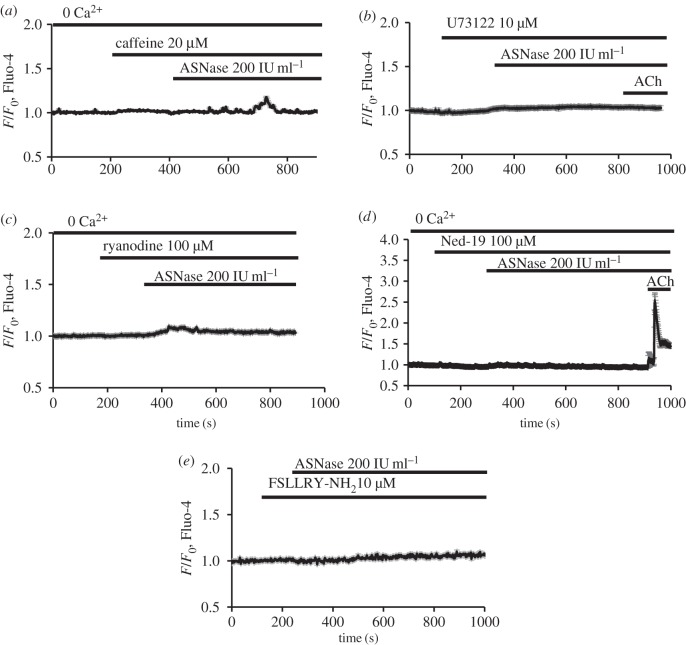
The primary intracellular Ca^2+^ release induced by asparaginase depends on IP_3_ and NAADP signalling mechanisms. The IP_3_R blocker caffeine (20 mM) inhibited very markedly the asparaginase-induced [Ca^2+^]_i_ elevations in the absence of external Ca^2+^ (*a*, averaged trace, *n* = 8). The PLC inhibitor U73122 (10 µM) blocked the asparaginase-induced [Ca^2+^]_i_ elevation in the absence of external Ca^2+^ (*b*, averaged trace, *n* = 11). ACh (1 µM) was applied at the end of each experiment and, as expected, did not elicit any change in [Ca^2+^]_i_ (*b*) as its effect depends on IP_3_ formation and IP_3_ receptors [23,24]. Ryanodine (100 µM) markedly inhibited the asparaginase-induced Ca^2+^ signals in the absence of external Ca^2+^ (*c*, averaged trace, *n* = 13). Ned-19 (100 µM) prevented the asparaginase-induced [Ca^2+^]_i_ elevation in the absence of external Ca^2+^ (*d*, averaged trace, *n* = 8). ACh (1 µM) was applied at the end of each experiment and, as expected, caused a major [Ca^2+^]_i_ rise as its effect does not depend on NAADP receptors [25,27]. The asparaginase-induced Ca^2+^ signals were virtually eliminated by the PAR2 inhibitor FSLLRY-NH_2_ (10 µM; *e*, averaged trace, *n* = 32).

To investigate the involvement of intracellular Ca^2+^ release channels in asparaginase-induced [Ca^2+^]_i_ elevations, we have used caffeine, a substance known to reliably inhibit IP_3_-mediated intracellular Ca^2+^ release in pancreatic acinar cells [23]. Caffeine (20 mM) substantially reduced the asparaginase-induced [Ca^2+^]_i_ elevations in a Ca^2+^-free solution (*n* = 8; figure 2*a*; compare with figure 1*e* as the appropriate control). The phospholipase C (PLC) inhibitor U73122 (10 µM) also significantly blocked the asparaginase-induced Ca^2+^ release as well as the response to 1 µM ACh (*n* = 11; figure 2*b*). Ryanodine (100 µM), inhibiting RyRs, also substantially reduced the asparaginase-induced [Ca^2+^]_i_ elevations (*n* = 13; figure 2*c*).

It has previously been shown that NAADP signalling in pancreatic acinar cells can be inactivated by the cell-permeable NAADP analogue, and selective antagonist, Ned-19 [27]. After pre-treatment of cells with 100 µM Ned-19, the asparaginase (200 IU ml^−1^)-induced [Ca^2+^]_i_ elevations were profoundly inhibited (virtually abolished; figure 2*d*, *n* = 8), whereas ACh (1 µM), eliciting Ca^2+^ release via IP_3_Rs independently of NAADP [25], was still able to evoke a typical [Ca^2+^]_i_ rise (*n* = 8; figure 2*d*).

### (d) Protease-activated receptor 2 is involved

PAR2 is widely expressed in human and animal tissues, including the pancreas, and has previously been implicated in the pathology of AP [53,54]. The activation of PAR family members is coupled to multiple heteromeric G proteins that lead to PLC activation and production of IP_3_ and diacylglycerol [55]. Therefore, we tested the possibility that asparaginase could activate PAR2 by pre-treating the cells with the PAR2 blocker FSLLRY-NH_2_ (10 µM) before the addition of asparaginase (200 IU ml^−1^). The PAR2 blocker reduced significantly (virtually abolished) the asparaginase-induced [Ca^2+^]_i_ oscillations as well as the sustained [Ca^2+^]_i_ elevation (figure 2*e*; *n* = 32).

### (e) Ca^2+^ extrusion mechanisms are affected by asparaginase

**Figure 3.**
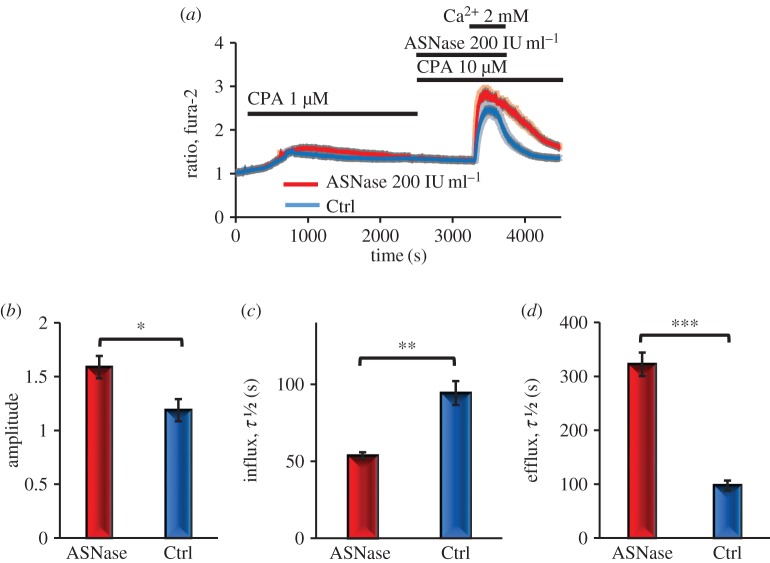
Asparaginase accelerates Ca^2+^ entry and substantially slows down Ca^2+^ extrusion. (*a*) In the absence of external Ca^2+^, CPA, a specific inhibitor of the Ca^2+^ pump in the ER membrane, causes a modest and largely transient [Ca^2+^]_i_ rise. When subsequently 2 mM Ca^2+^ is added to the external solution, there is a marked rise in [Ca^2+^]_i_, which then declines after removal of external Ca^2+^. In the presence of asparaginase (red averaged trace, *n* = 32), the amplitude (*b*) and the rate of rise of [Ca^2+^]_i_ (*c*) are somewhat increased (*p* < 0.048 and *p* < 0.0001, respectively) when compared with control (*a*, blue averaged trace, *n* = 34). The extrusion of Ca^2+^ by the plasma membrane Ca^2+^ pumps, observed as the decline in [Ca^2+^]_i_ following removal of external Ca^2+^, is very markedly and significantly (*p* < 0.0001) reduced in the presence of asparaginase (*a*,*d*). (Online version in colour.)

To study Ca^2+^ movements in more detail, we have applied a specific protocol routinely used to assess Ca^2+^ entry and extrusion [9,38]. In these experiments, Ca^2+^ stores were emptied using the ER Ca^2+^ pump inhibitor cyclopiazonic acid (CPA) in a nominally Ca^2+^-free solution. Thereafter, 2 mM of Ca^2+^ was added to the external solution for a short period and then removed (figure 3*a*). In the presence of asparaginase, the Ca^2+^ entry following external Ca^2+^ admission was significantly increased (figure 3*a*), assessed by both amplitude of the [Ca^2+^]_i_ change (figure 3*b*) and the rate of [Ca^2+^]_i_ increase (half-time of the increase, figure 3*c*). However, the quantitatively most important effect of asparaginase was to slow down the rate of Ca^2+^ extrusion after removal of external Ca^2+^ (figure 3*a*). The half-time of [Ca^2+^]_i_ recovery was more than three times longer than in the control cells (figure 3*d*).

### (f) Asparaginase depletes intracellular adenosine triphosphate

**Figure 4.**
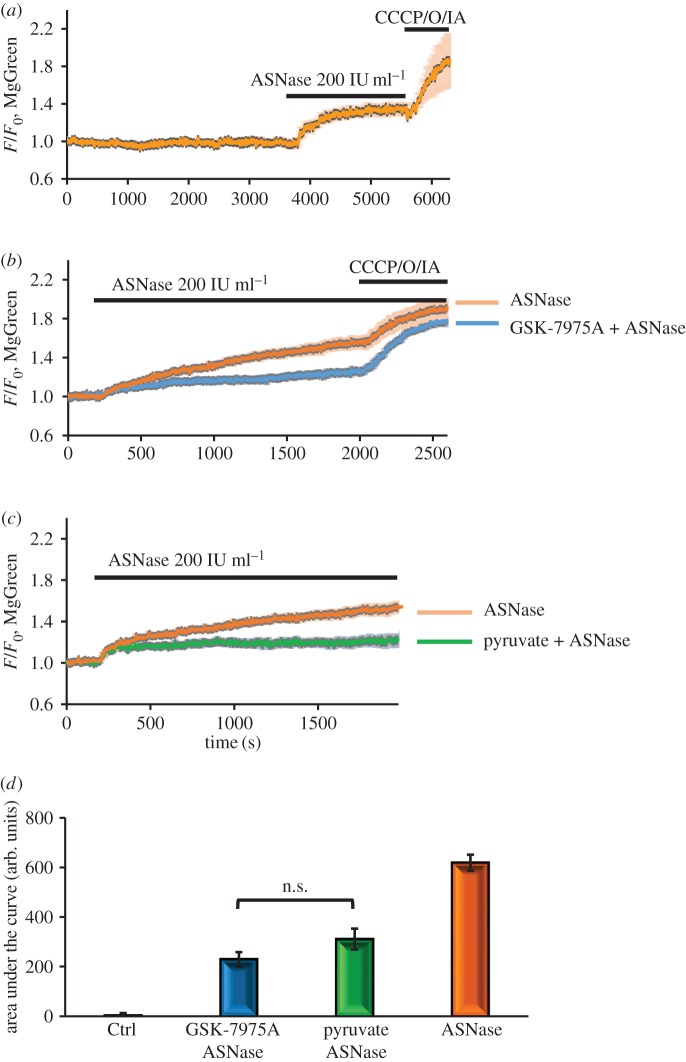
Asparaginase reduces intracellular ATP levels as assessed by increases in [Mg^2+^]_i_. In these experiments, changes in [Mg^2+^]_i_ were assessed by monitoring changes in MgGreen fluorescence. Most of the Mg^2+^ inside cells is bound to ATP, so when ATP declines—for example owing to interference with mitochondrial function—[Mg^2+^]_i_ will increase. A rise in [Mg^2+^]_i_ can therefore be taken to represent a reduction in the intracellular ATP level [58]. Asparaginase (200 IU ml^−1^) markedly increased [Mg^2+^]_i_ and a further rise occurred after poisoning mitochondrial function with a cocktail of CCCP (5 nM), oligomycin (10 µM) and sodium iodoacetate (2 mM; *a*, averaged trace, *n* = 39). The asparaginase effect was markedly reduced by GSK-7975A (*b*: orange averaged trace, *n* = 21; blue averaged trace, *n* = 39) and was also markedly reduced by supplementing the external medium with pyruvate (1 mM; *c*, *n* = 8). Comparisons of the integrated responses (‘areas under the curve’ from the start of the responses until 1800 s later) show that GSK-7975A and pyruvate significantly reduced the asparaginase-induced ATP depletion (*p* < 0.0001 for both treatments).

Ca^2+^ extrusion is an energy-demanding process and has previously been found to be abnormal in pancreatic acinar cell pathologies owing to disruption of mitochondrial metabolism and, therefore, reduction of ATP levels [42]. We have conducted indirect assessments of intracellular changes in ATP concentration, using Magnesium Green (MgGreen) fluorescence measurements. As most of the intracellular ATP will be in the form of Mg-ATP, a reduction of the ATP concentration will increase the fluorescence intensity of MgGreen owing to the inevitable increase of [Mg^2+^]_i_ [56–58]. By this measure, asparaginase induced a substantial reduction of intracellular ATP levels (figure 4*a*) superseded only by the full ATP depletion caused by a mixture of the protonophore CCCP, oligomycin and iodoacetate [42].

Because the presence of external Ca^2+^ was important for the cytoplasmic and mitochondrial effects induced by asparaginase, we decided to check if inhibition of Ca^2+^ entry channels [9,43] could affect the ATP loss evoked by asparaginase. Blocking Ca^2+^ entry by GSK-7975A (10 µM) substantially reduced the ATP loss evoked by asparaginase (figure 4*b*).

### (g) The effect of pyruvate

It has been shown previously that ethyl pyruvate (aliphatic ester derived from pyruvic acid [59]) attenuates the severe AP induced by sodium taurocholate in rats [59]. When we included 1 mM pyruvate in the external solution, we found that the asparaginase-induced ATP loss was substantially reduced compared with control experiments (figure 4*c*,*d*). The protective effect was significant and similar to what was achieved by GSK-7975A (figure 4*b*,*d*), although it did not give complete protection.

### (h) Necrosis

**Figure 5.**
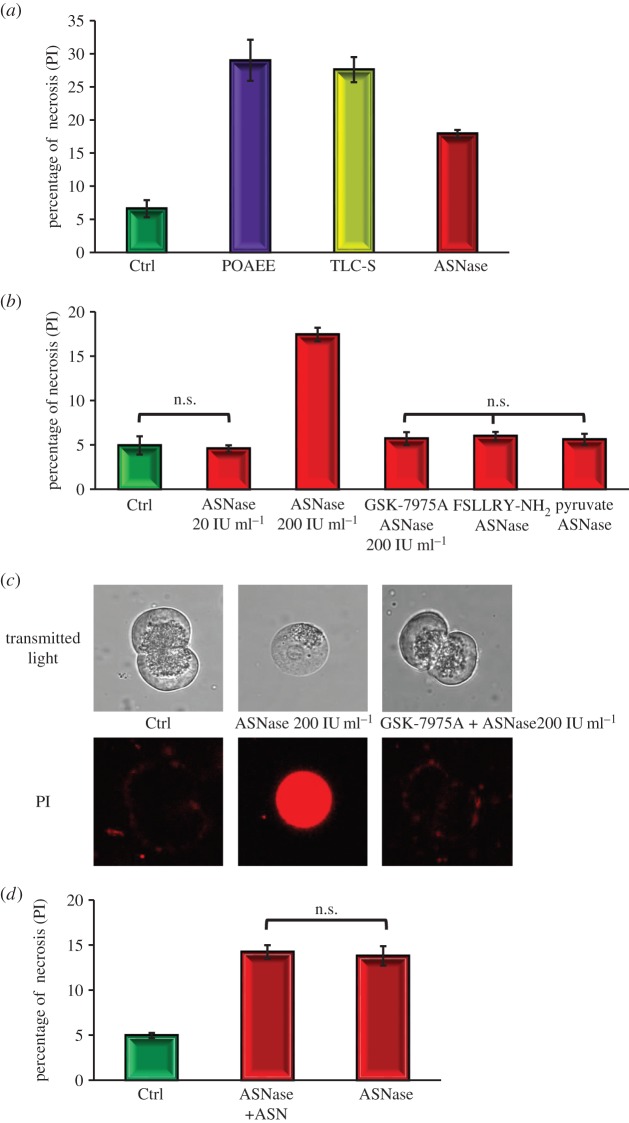
Asparaginase induces necrosis in pancreatic acinar cells. (*a*) The level of necrosis induced by 200 IU ml^−1^ of asparaginase (17.4 ± 0.4%, eight series with *n* > 250) is comparable with, but somewhat lower than, that caused by the alcohol metabolite POAEE (100 µM; 29 ± 3.1%, three series of experiments with *n* > 100) or the bile acid TLC-S (200 µM; 27.6 ± 1.9%, three series of experiments with *n* > 120), whereas a lower concentration of asparaginase (20 IU ml^−1^) did not induce any significant change in the level of necrosis (*b*) when compared with control (4.5 ± 0.4 and 4.5 ± 0.7%, respectively, *p* > 0.8, each test had eight series of experiments with *n* > 130 of tested cells in each group). The CRAC channel inhibitor GSK-7975A (10 µM) essentially abolished asparaginase-induced necrosis to a level not significantly different from control (*p* > 0.24), while significantly (*p* < 0.0001, eight series, *n* > 70) lower than that caused by 200 IU ml^−1^ of asparaginase alone (*b*). The PAR2 inhibitor FSLLRY-NH_2_ (10 µM) significantly blocked the asparaginase-induced necrosis (*p* < 0.0001; four series of experiments with *n* > 150 cells in each sample). Pyruvate (1 mM) reduced the asparaginase-induced necrosis to near the control level (*b*; *p* < 0.0001, three series of experiments with the number of tested cells in each group more than 130). (*c*) Representative images of cells from some of the experiments shown in (*b*). Transmitted light images (upper row) and PI-stained fluorescence images (lower row). (*d*) The presence or absence of asparagine (50 µM) made no difference to the level of asparaginase-induced necrosis (*p* > 0.75, three series of experiments with more than 70 cells in each sample).

We have previously shown that the cytosolic Ca^2+^ overload and ATP deprivation induced by fatty acid ethyl esters and bile acids lead to necrosis [38], and we have therefore now tested whether asparaginase can also induce necrosis, the hallmark of AP [33,38]. The extent of necrosis induced by asparaginase (200 IU ml^−1^) treatment (17.4 ± 0.4% of the cells; figure 5*a*) was comparable to, but somewhat smaller than, the level of necrosis induced by pamitoleic acid ethyl ester (POAEE; 29 ± 3.1%) or the bile acid taurocholic acid sulphate (TLC-S; 27.6 ± 1.9%; figure 5*a*), whereas a lower concentration of asparaginase (20 IU ml^−1^) did not increase the level of necrosis above that seen in control experiments (figure 5*b*). The CRAC channel inhibitor GSK-7975A (10 µM) [9,43] reduced the asparaginase-induced necrosis to the control level (4.5 ± 0.7%; figure 5*b*). Pyrazole compounds have generally been thought to inhibit other types of cation channels, namely the relatively non-selective TRP (transient receptor potential) channels, which have significant Ca^2+^ permeability [60,61], but pyrazole6 (Pyr6) has been shown to have more of an effect on the very Ca^2+^-selective CRAC channels [61], which are the ones specifically inhibited by GSK-7975A and CM-128 [9,43]. In our experiments, Pyr6 partially inhibited asparaginase-induced necrosis to 8.4 ± 0.9%. Both caffeine and Ned-19 inhibited asparaginase-induced necrosis to control levels (4.2 ± 0.5 and 6.6 ± 1.2%, respectively). A PAR2 inhibitor (FSLLRY-NH_2_) significantly blocked asparaginase-induced necrosis (figure 5*b*). We also tested the effect of pyruvate on asparaginase-induced necrosis. As seen in figure 5*b*, this gave significant protection against necrosis. Figure 5*c* shows representative images of some of the cells under the treatment protocols, together with the results of staining for propidium iodide (PI). It is seen that asparaginase (200 IU ml^−1^) elicited strong intracellular PI staining and that GSK-7975A provided protection against this.

Asparaginase kills lymphoblastic cells by depriving them of asparagine, which they—unlike normal cells—cannot produce themselves [62]. The effects of asparaginase on normal pancreatic acinar cells described in this study are therefore unlikely to be owing to asparagine deprivation. We tested whether there was any difference between the ability of asparaginase to induce necrosis in the absence or presence of asparagine. As seen in figure 5*d*, there was no difference in the necrosis levels evoked by asparaginase in the presence or absence of asparagine.

## 3. Discussion

**Figure 6.**
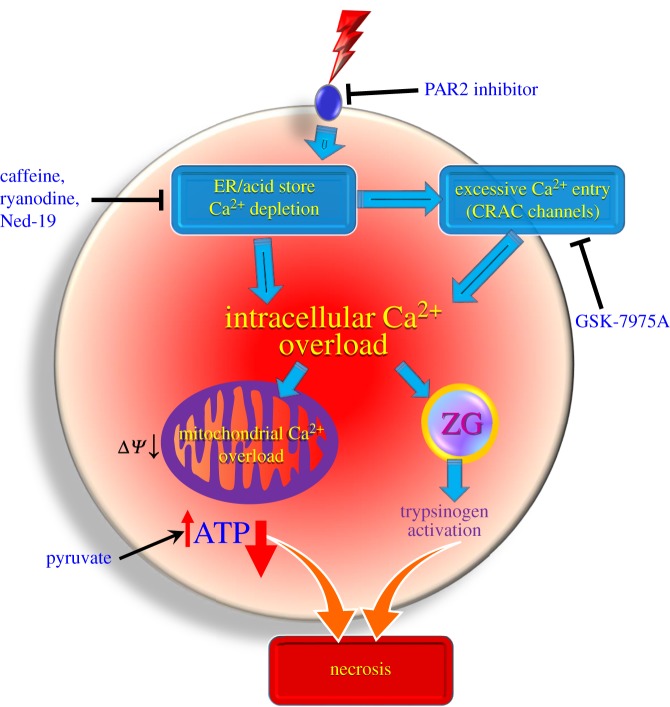
Schematic diagram illustrating the likely effects of asparaginase on pancreatic acinar cells, based partly on the new data shown in this study and partly on analogy with processes identified in previous studies on the mechanisms by which fatty acid ethyl esters and bile acids cause AP [38]. Potential sites for therapeutic intervention are also indicated.

The results presented here, on the asparaginase-elicited injury to pancreatic acinar cells, provide fresh evidence for the hypothesis that all types of AP are owing to toxic Ca^2+^ signal generation and explain how asparaginase could cause AP (figure 6). Asparaginase, like fatty acid ethyl esters and bile acids, can evoke sustained [Ca^2+^]_i_ elevation owing to release of Ca^2+^ from intracellular stores followed by store-operated Ca^2+^ entry through CRAC channels. Qualitatively, the effects of asparaginase fit well with those induced by fatty acid ethyl esters and bile acids, which we have described previously [33,38]. However, the sustained [Ca^2+^]_i_ elevations evoked by asparaginase are somewhat smaller than those evoked by bile acids or fatty acid ethyl esters. Nevertheless asparaginase evokes significant reductions in the intracellular ATP levels and extensive necrosis. Further studies on mitochondrial Ca^2+^ handling during the action of asparaginase are warranted because the regulation of mitochondrial Ca^2+^ uptake under different conditions may be a critical issue [63,64].

The findings presented in this study provide the first mechanistic insights into the process by which asparaginase treatment of ALL may cause AAP (figure 6). These insights also provide the first pointers to rational therapies (figure 6) that may prevent the currently necessary cessation of asparaginase treatment of ALL in cases of severe pancreatitis. The most accessible therapeutic target is the Ca^2+^ entry route, namely the previously characterized CRAC channels [9,43,44,65]. We have now shown that the asparaginase-induced Ca^2+^ elevations depend on CRAC-mediated Ca^2+^ entry and, therefore, are strongly inhibited by the CRAC channel inhibitor GSK-7975A and also that, consequently, asparaginase-induced necrosis is dramatically reduced to near control levels by GSK-7975A (figure 5*b*). On the basis of the previously documented protective effects of Ca^2+^ entry channel inhibition against alcohol-related pancreatic pathology in isolated cell clusters [9,38], the recent confirmation of its effectiveness *in vivo* in three different mouse pancreatitis models [43] and the very recently demonstrated inhibition of prolonged Ca^2+^ signal generation in pancreatic stellate cells [31,32], our new data indicate that this therapeutic approach is also likely to be successful against asparaginase-induced pancreatitis. Clearly, the next—but challenging—step would be to test the effectiveness of CRAC channel blockade against AAP in an *in vivo* mouse model.

The mechanism by which asparaginase induces pancreatitis is fundamentally different from its therapeutic action on the lymphoblastic cells in ALL. The asparaginase effect on cancer cells relies on depletion of asparagine, which the malignant cells cannot produce themselves, in contrast to normal cells [62], whereas the side-effect of asparaginase, inducing pancreatitis, is owing to activation of a signal transduction mechanism involving PAR2, the intracellular messengers IP_3_ and NAADP, and the intracellular receptors IP_3_Rs, RYRs and possibly TPCs (figure 6). The asparaginase effect on the pancreas is therefore independent of the presence or absence of asparagine (figure 5*d*). This means that there are several potential intervention points available for treating the side-effect of asparaginase (figure 6), without interfering with its primary effect on the cancer cells. The primary site of action of asparaginase on pancreatic acinar cells seems to be PAR2. This receptor has previously been implicated in AP, although its exact role is still debated [53,54]. Blocking PAR2 in our experiments inhibited both the pathological [Ca^2+^]_i_ elevations (figure 2*e*) and the asparaginase-induced necrosis (figure 5*b*), suggesting that PAR2 inhibitors in addition to, or in combination with, CRAC channel inhibitors could be a useful tool to supplement asparaginase ALL treatment in AAP cases.

Both Ca^2+^ entry and extrusion are significantly affected by asparaginase, leading to formation of the pathological elevated [Ca^2+^]_i_ plateau, and this sustained elevation of [Ca^2+^]_i_ would appear to be responsible for the necrosis. The demonstrated reduction of the intensity of Ca^2+^ extrusion (figure 3*a*) is clearly a key element, and the simplest explanation for this is the reduction in the intracellular ATP level (figure 4*a*) limiting the energy supply to the Ca^2+^ ATPase in the plasma membrane. When energy supply is partially restored by the addition of pyruvate (figure 4*d*), it provides a similar degree of protection against pancreatic necrosis to PAR2 inhibition or GSK-7975A (figure 5*b*). Clearly, both Ca^2+^ and ATP play key roles in pancreatic pathology, as indeed they do in physiological regulation of secretion, and therapeutic strategies must take both into account.

## 4. Material and methods

### (a) Materials

All fluorescent dyes were purchased from ThermoFisher Scientific (Invitrogen, UK), and CPA was from Merck Millipore (Calbiochem, UK). Collagenase was obtained from Worthington (USA), asparaginase was from Abcam (UK), the PAR2 inhibitor FSLLRY-NH2 from TOCRIS (UK) and GSK-7975A from GlaxsoSmithKline (UK). All other chemicals were purchased from Sigma. C57BL/6 J mice were from Charles River UK Ltd.

### (b) Isolation of pancreatic acinar cells

Pancreatic acinar cells were isolated as previously described [9]. Briefly, animals were sacrificed according to the Animal Scientific Procedures Act, 1986 and approved by the Ethical Review Committee of Cardiff University. After dissection, the pancreas was digested using collagenase-containing solution (200 U ml^−1^, Worthington, UK) and incubated in a 37°C water bath for 14–15 min. The extracellular solution contained: 140 mM NaCl, 4.7 mM KCl, 10 mM Hepes, 1 mM MgCl_2_, 10 mM glucose, pH 7.2, and CaCl_2_ (0–2 mM as described in the text).

### (c) Fluorescence measurements

For measurements of [Ca^2+^]_i_, isolated pancreatic acinar cells were loaded with Fluo-4-AM (5 µM; excitation 488 nm) or Fura-2-AM (2.5 µM; excitation 365 and 385 nm) following the manufacturer's instruction. The cells were then washed and re-suspended in extracellular solution containing 1 mM CaCl_2_.

Measurement of intracellular ATP was performed with MgGreen, which senses changes in [Mg^2+^]_i_ at concentrations around the resting [Mg^2+^]_i_ (approx. 0.9 mM). This was used as an indirect approach to detect cytosolic ATP depletion [58]. Pancreatic acinar cells were incubated with 4 µM MgGreen for 30 min at room temperature (excitation 488 nm). ATP depletion mixture (4 µM CCCP, 10 µM oligomycin and 2 mM iodoacetate) was applied for a final 10 min to induce maximum ATP depletion [42].

Necrotic cell death was assessed with PI uptake as previously described [9].

All experiments were performed at room temperature using freshly isolated cells attached to coverslips of the perfusion chamber. Fluorescence was imaged over time using an Leica TCS SPE confocal microscope.

### (d) Statistical analysis

Data are presented as mean ± SEM. Statistical significance and *p-*values were calculated using *t*-test or ANOVA, with *p* < 0.05 considered significant.

## Data accessibility

Data sets for this article have been obtained and processed using Leica LAS AF software (http://www.leica-microsystems.com/products/microscope-software/) needed to open the original files. Supporting files will be made available in the Research section via: https://www.cardiff.ac.uk/people/view/108900-gerasimenko-oleg.
